# Neuroimaging in FDXR-related mitochondriopathy

**DOI:** 10.1007/s00247-026-06734-8

**Published:** 2026-07-22

**Authors:** Allison Lam, Bryce D. Beutler, Siddhanth S. Hegde, Matthew J. Barkovich, Anthony R. Zamary, Fernando A. Torres

**Affiliations:** 1 Kaiser Permanente Bernard J. Tyson School of Medicine, Los Angeles, CA USA; 2 Department of Radiology, University of Southern California, Keck School of Medicine, 1500 San Pablo Street, 2nd Floor, Los Angeles, CA 90033 USA; 3 Department of Radiology, University of California, San Francisco, San Francisco, CA USA; 4 Department of Radiology, Los Angeles Medical Center, Southern California Permanente Medical Group, Los Angeles, CA USA

**Keywords:** Atrophy, Child, Ferredoxins, Neuroimaging, Optic nerve, Oxidative stress, Oxidoreductases, Preschool, White matter

## Abstract

Ferredoxin reductase (FDXR)-related mitochondriopathy is a rare autosomal recessive disorder caused by biallelic pathogenic variants in the FDXR gene. The clinical presentation is highly variable, ranging from isolated sensory neuropathies to severe infantile-onset encephalopathy with early demise. Neuroimaging is typically normal. However, oxidative stress in the setting of systemic infection can precipitate severe symptoms with distinct imaging manifestations, including confluent white matter signal abnormality, longitudinally extensive spinal cord lesions, and optic nerve enhancement. We describe a previously healthy and asymptomatic 2-year-old female with genetically confirmed FDXR-related mitochondriopathy who was found to have a distinct pattern of diffusion restriction, rapid parenchymal atrophy, and optic nerve changes following a respiratory illness. Early recognition and management of this rare but potentially devastating entity can help prevent vision loss and improve functional outcomes.

## Introduction

Ferredoxin reductase (FDXR)-related mitochondriopathy is a rare autosomal recessive disorder caused by biallelic pathogenic variants in the FDXR gene. The disorder is characterized by a variable phenotype with optic nerve atrophy being the most distinct and consistent feature; developmental delay, hypotonia, ataxia, and sensorineural hearing loss may occur with variable severity and frequency [1–4].The imaging features of FDXR-related mitochondriopathy have been incompletely characterized, with most reported cases showing either a normal brain MRI or nonspecific findings, such as cerebral volume loss and optic nerve atrophy [1, 2].

We present a case of genetically confirmed FDXR-related mitochondriopathy demonstrating the evolution from normal neuroimaging to Leigh syndrome-like basal ganglia involvement that was precipitated by a viral respiratory infection.

## Case report

A 4-month-old Hispanic female born at 38 weeks and 3 days presented for evaluation of gross motor delay and abnormal eye movements. The parents reported brief episodes of downward gaze lasting for a few seconds at a time. Ophthalmologic evaluation showed intermittent slow horizontal pendular eye movements with brief episodes of downbeat nystagmus.

A contrast-enhanced MRI of the brain and orbits demonstrated no abnormality. The patient returned at 6 months of age. Neurologic examination revealed new central hypotonia, impaired balance, and diminished strength. Correlation of the clinical history and neurologic examination raised concern for an underlying neurogenetic or metabolic disorder, prompting comprehensive genetic testing. Whole exome sequencing subsequently identified a homozygous pathogenic missense variant, c.1156C>T (p.R386W), in the *FDXR* gene, confirming the diagnosis of FDXR-related mitochondrial disease (OMIM 619333). Both parents were confirmed heterozygous carriers.

Neurologic symptoms gradually progressed between 6 months and 2 years of age, characterized by progressive feeding intolerance, poor weight gain, constipation, urinary retention, and progressive visual decline. Ophthalmologic examinations revealed severe, progressive optic nerve atrophy, resulting in complete blindness by 2 years of age. However, repeat MRI examinations of the brain during this interval remained normal. The patient developed new symptoms between 2 years and 2.5 years of age, including sleep disturbances, irritability, and episodes concerning for seizure activity.

At 2.5 years of age, the patient was hospitalized with acute respiratory failure following sequential COVID-19 and human metapneumovirus (HMPV) infections complicated by secondary methicillin-susceptible *Staphylococcus aureus* (MSSA) pneumonia. Intubation was required on the first hospital day for escalating respiratory distress and hypoxemia. During the hospitalization, the patient demonstrated marked neurologic decline, characterized by progressive hypotonia, diminished deep tendon reflexes, and complete loss of the gag reflex. Differential considerations included hypoxic-ischemic injury, acute ischemic infarct, and toxic-metabolic encephalopathy. An MRI of the brain with and without contrast was acquired to assess for an underlying cause of the rapidly worsening neurologic status.

**Fig. 1:**
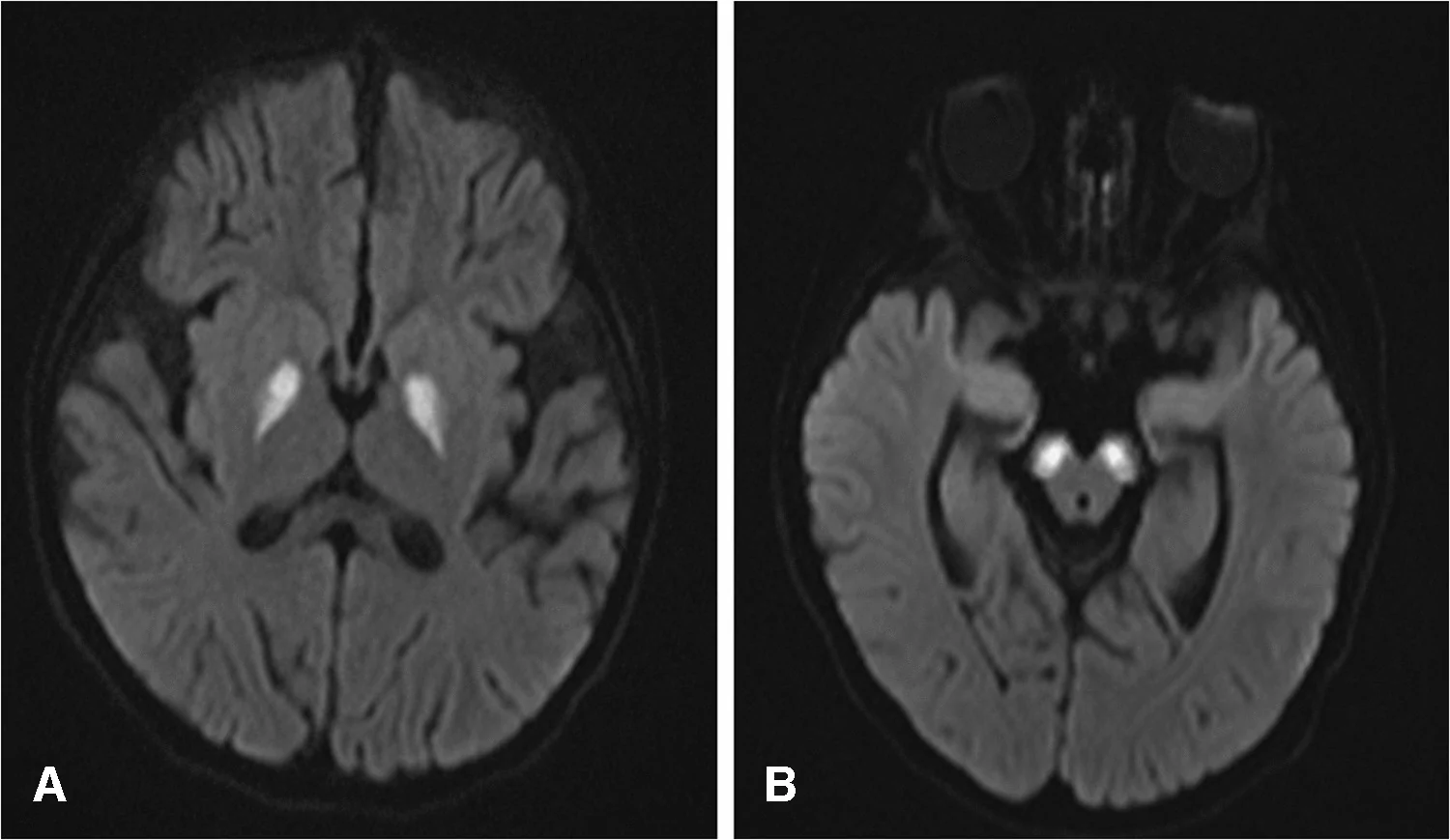
An MRI of the brain with and without contrast was ordered to evaluate for an underlying cause of progressively worsening neurologic status in a 2.5-year-old female with genetically-confirmed FDXR-related mitochondriopathy. **A** Axial diffusion-weighted images (DWI; *b* = 1,000 s/mm^2^) demonstrate a striking symmetric pattern of restricted diffusion involving the bilateral globi pallidi interna and externa. **B** Axial DWI acquired inferiorly revealed contiguous involvement extending inferiorly through the cerebral peduncles to involve the substantia nigra of the midbrain. Corresponding signal abnormality is present on accompanying T2/FLAIR sequences, consistent with acute cytotoxic edema affecting metabolically vulnerable deep gray nuclei and brainstem structures. The distribution and symmetry of involvement produce a Leigh-like imaging pattern characteristic of mitochondrial metabolic decompensation rather than typical hypoxic-ischemic injury. Notably, MRI examinations obtained prior to the patient’s acute respiratory illness were normal, indicating interval development of these abnormalities in the setting of severe systemic infection and respiratory failure

**Fig. 2:**
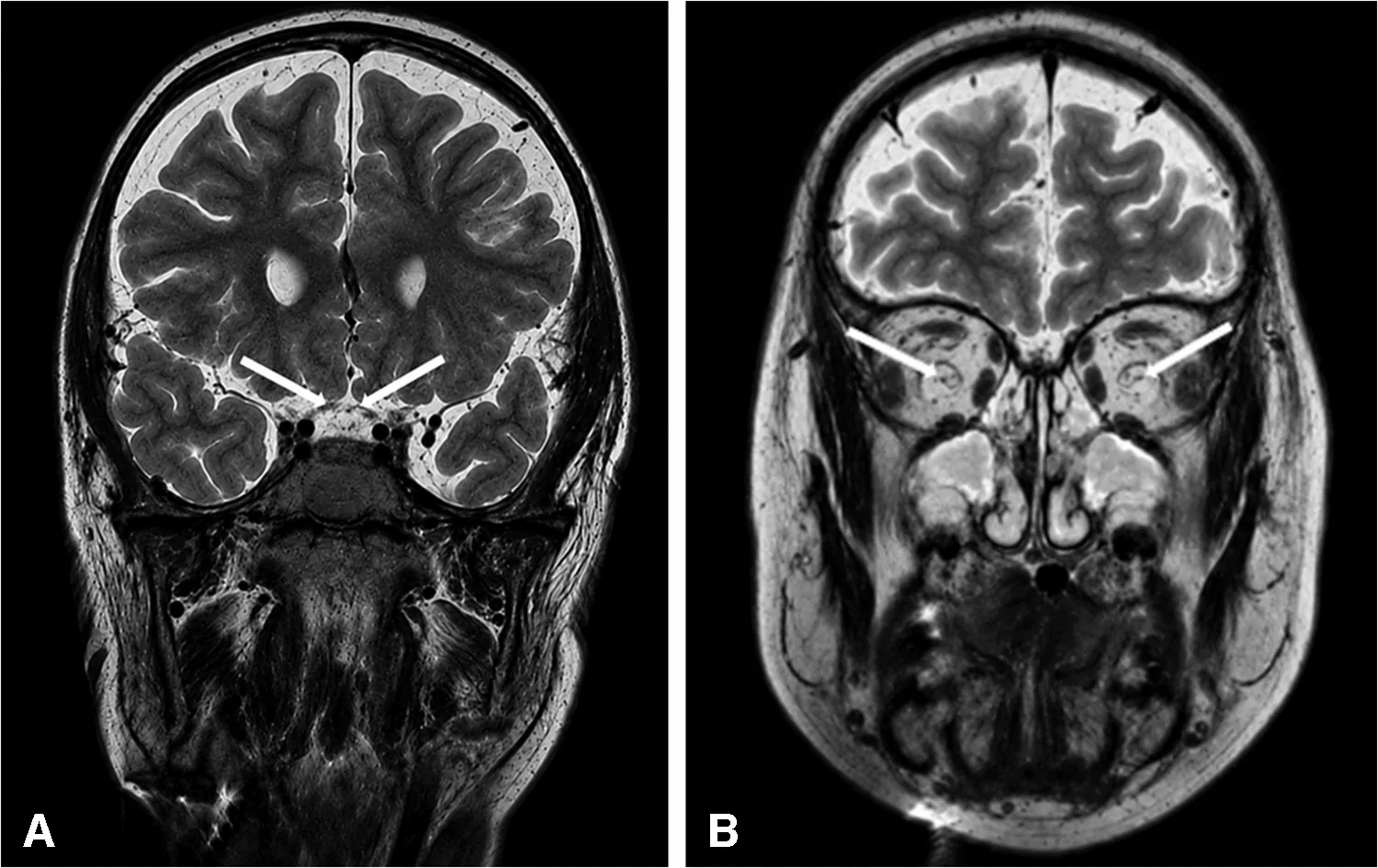
An MRI of the brain with and without contrast was ordered to evaluate for an underlying cause of progressively worsening neurologic status in a 2.5-year-old female with genetically-confirmed FDXR-related mitochondriopathy. **A** Coronal T2-weighted images demonstrate severe bilateral optic pathway atrophy, including marked thinning of the prechiasmatic optic nerves (*arrows*). **B** Coronal T2-weighted images also showed diffuse atrophy of the intraorbital optic nerves with associated relative prominence and cerebrospinal fluid distention of the optic nerve sheaths, producing an appearance of disproportionate sheath enlargement surrounding the markedly attenuated nerves (*arrows*). The pronounced optic nerve atrophy with accompanying sheath distention represents a characteristic imaging manifestation of FDXR-related mitochondrial disease and may help distinguish this entity from other mitochondrial encephalopathies and inherited optic neuropathies

The MRI showed a striking symmetric pattern of restricted diffusion with associated T2/FLAIR hyperintensity involving the globi pallidi and extending inferiorly through the cerebral peduncles into the substantia nigra of the midbrain (Fig. 1). Additional findings included marked atrophy of the optic nerves, optic chiasm, and optic tracts (Fig. 2), as well as moderate diffuse cerebral volume loss that was new or markedly progressed compared with MRI performed 6 months earlier. Although hypoxic-ischemic injury was initially considered in the setting of respiratory failure, the distribution of cytotoxic edema was atypical for global hypoxic injury, as there was sparing of the perirolandic cortices and no evidence of cortical ribboning. The pronounced involvement of the globi pallidi and substantia nigra instead favored a Leigh-like pattern of selective involvement of metabolically vulnerable deep gray nuclei and brainstem structures seen in mitochondrial encephalopathies. The imaging findings were therefore interpreted in the context of acute metabolic decompensation triggered by severe infection in the setting of genetically confirmed FDXR-related mitochondrial disease.

The patient was ultimately discharged after a 56-day hospitalization, which entailed predominantly supportive care and antibiotics. A follow-up examination performed 2 months after hospitalization showed improved neurologic function, as evidenced by return of normal deep tendon reflexes and decreased dysphagia. However, the patient continues to demonstrate profound developmental regression and remains dependent on tracheostomy and gastrostomy tube support (Supplemental Table 1).

**Table 1:** Imaging features of FDXR-related mitochondriopathy compared with other mitochondrial disorders

Feature	FDXR-related mitochondriopathy	Leigh syndrome	POLG-related disorders	MELAS	LBSL
White matter	Confluent, symmetric	Mild/late	Variable	Minimal	Confluent, tract-specific
Optic nerve	Prominent involvement (key feature)	Occasional	Rare	Rare	Uncommon
Spinal cord	Longitudinally extensive lesions	Possible	Rare	Rare	Prominent, tract-based
Basal ganglia	Variable	Classic involvement	Variable	Variable	Variable
Brainstem	Variable	Characteristic	Variable	Rare	Tract involvement
Cortex	Rare	Rare	Prominent (stroke-like)	Prominent (stroke-like)	Rare
Diffusion restriction	Variable	Common	Common (cortical)	Common	Variable
Enhancement	Rare	Rare	Variable	Variable	Rare
MR spectroscopy	±lactate	Lactate peak	Lactate may be present	Lactate peak in both normal and abnormal brain parenchyma	Lactate peak
Key imaging clue	White matter+optic nerve+cord	Basal ganglia+brainstem	Cortical stroke-like lesions	Migratory cortical lesions	Tract-specific white matter+cord

*FDXR*, ferredoxin reductase; *LBSL*, leukoencephalopathy with brainstem and spinal cord involvement and lactate elevation; *MELAS*, mitochondrial encephalomyopathy, lactic acidosis and stroke-like episodes; *POLG*, DNA polymerase gamma

## Discussion

### FDXR-related mitochondriopathy

FDXR-related mitochondriopathy is an autosomal recessive disorder caused by biallelic pathogenic variants in the *FDXR* gene, which encodes ferredoxin reductase, a mitochondrial flavoprotein located in the inner mitochondrial membrane [1]. Fewer than 50 cases have been reported worldwide [1–3]. The clinical spectrum ranges from isolated sensorial neuropathies to severe infantile-onset encephalopathy with early demise [2, 4].

The clinical phenotype of FDXR-related mitochondriopathy is highly variable. Optic nerve atrophy is the most consistent feature and may be present in up to 89% of patients [3]. Notably, optic nerve atrophy represents a common downstream consequence of mitochondrial dysfunction and is not specific to FDXR-related disease, as similar findings are observed across multiple mitochondrial optic neuropathies, including Leber hereditary optic neuropathy and dominant optic atrophy.

The neuroimaging features of FDXR-related mitochondriopathy have been incompletely described in the literature. Individuals with genetically-confirmed FDXR-related mitochondriopathy have been shown to exhibit either a normal brain MRI or nonspecific findings, such as cerebral atrophy and optic nerve atrophy [1, 2]. Limited data and case series describe severe optic nerve atrophy, confluent white matter signal abnormality, and longitudinally extensive spinal cord lesions. To the best of our knowledge, our case is the first in which neuroimaging findings evolved from essentially normal to a distinct pattern of pronounced diffusion restriction following a viral illness. Additional imaging features seen in our case included marked atrophy of the optic nerves, chiasm, and tracts and global brain parenchymal volume loss, reflecting progressive neurodegeneration.

The clinical and imaging features of FDXR-related mitochondriopathy may mimic those of other mitochondriopathies, including Leigh syndrome, POLG-related disorders, mitochondrial encephalomyopathy, lactic acidosis, and stroke-like episodes (MELAS), and leukoencephalopathy with brainstem and spinal cord involvement and lactate elevation (LBSL) (Table 1).

### Leigh syndrome

The hallmark neuroimaging feature of Leigh syndrome is symmetric T2/FLAIR-hyperintense signal involving the putamina with relative sparing of the caudate nuclei. Diffusion restriction may be present in the hyperacute or acute phase. Progressive disease is characterized by T2/FLAIR signal abnormality extending to the globi pallidi, thalami, periaqueductal gray matter, tectal plate, and dorsal pons and pontomedullary junction. Cavitation can be seen in advanced cases. The imaging findings in the appropriate clinical context are relatively specific. However, MR spectroscopy may be of value in equivocal cases and will classically reveal a markedly elevated lactate peak affecting the basal ganglia or brainstem. Genetic testing is required for confirmation [5].

### POLG-related disorders

The neuroimaging manifestations of POLG-related disorders vary based on the specific phenotype, which is determined by a combination of mutation-specific effects on mitochondrial DNA maintenance, inherited allelic combinations, and tissue vulnerability to mitochondrial dysfunction. Alpers-Huttenlocher syndrome is classically characterized by a distinct pattern of T2/FLAIR signal abnormality involving the occipital cortices and thalami. Alpers-Huttenlocher syndrome is also associated with liver failure, and thus symmetric subthalamic T1 shortening involving the globi pallidi, a known manifestation of hepatic encephalopathy, is commonly observed. Neuroimaging features of adult-onset POLG-related disorders are often more subtle and characterized by slowly progressive cerebellar-predominant neurodegeneration rather than acute lesions [6].

### MELAS

The neuroimaging features of MELAS are unique among other mitochondriopathies. Acute-phase findings include cortical and subcortical expansile T2/FLAIR-hyperintense lesions in a predominantly parieto-occipital and parieto-temporal distribution. However, MELAS demonstrates a distinct migratory pattern, characterized by lesion migration and evolution over days to weeks, leaving behind cortical laminar necrosis following resolution. In addition, in contrast to cytotoxic edema seen in acute arterial infarcts, the cortical and subcortical lesions in MELAS typically demonstrate enhancement and hyperperfusion, reflecting dynamic reperfusion hyperemia. The deep white matter is nearly always spared in MELAS, which helps distinguish it from ischemic processes. MR spectroscopy in MELAS is also unique, showing elevated lactate in both affected and normal brain parenchyma [7].

### LBSL

The neuroimaging features of LBSL are virtually pathognomonic. A diagnosis can be established in the appropriate clinical context if the patient demonstrates a unique pattern of T2/FLAIR-hyperintense signal involving the periventricular and deep white matter, dorsal columns and lateral corticospinal tracts, and pyramids and/or decussation of the medial lemniscus. Diffusion restriction may be present along the margins of the white matter signal abnormality. The presence of profound cerebral atrophy in infancy with tortuous cortical vessels is also suggestive of underlying LBSL, but somewhat nonspecific [8].

### Treatment

Treatment for FDXR-related mitochondriopathy remains largely supportive. Regular ophthalmologic monitoring for progressive optic atrophy and retinopathy, audiology assessment for sensorineural hearing loss, and neurologic follow-up for developmental regression and seizures are recommended [1–3].

In conclusion, this case highlights the importance of recognizing FDXR-related mitochondriopathy as a cause of Leigh-like basal ganglia involvement and documents the potential for infection-triggered acute neurologic decompensation with evolution from normal to abnormal neuroimaging. Serial neuroimaging may be valuable in patients with FDXR deficiency, particularly during acute illnesses, as normal baseline imaging does not exclude the possibility of future development of cytotoxic edema and neurodegeneration.

## Data Availability

No datasets were generated or analysed during the current study.

## References

[1] Peng Y, Shinde DN, Valencia CA et al (2017) Biallelic mutations in the ferredoxin reductase gene cause novel mitochondriopathy with optic atrophy. Hum Mol Genet 26:4937–4950 10.1093/hmg/ddx377 PMC5886230 29040572

[2] Stenton SL, Piekutowska-Abramczuk D, Kulterer L et al (2021) Expanding the clinical and genetic spectrum of FDXR deficiency by functional validation of variants of uncertain significance. Hum Mutat 42:310–319 10.1002/humu.24160 33348459

[3] Kaler A, Couser N (2025) Ocular and neurological manifestations of the FDXR-related disorder. J AAPOS 29:104102 10.1016/j.jaapos.2024.104102 39746537

[4] Paul A, Drecourt A, Petit F et al (2017) FDXR mutations cause sensorial neuropathies and expand the spectrum of mitochondrial Fe-S-synthesis diseases. Am J Hum Genet 101:630–637 10.1016/j.ajhg.2017.09.007 PMC5630197 28965846

[5] Ruhoy IS, Saneto RP (2014) The genetics of Leigh syndrome and its implications for clinical practice and risk management. Appl Clin Genet 7:221–234 10.2147/TACG.S46176 PMC4235479 25419155

[6] Rahman S, Copeland WC (2019) POLG-related disorders and their neurological manifestations. Nat Rev Neurol 15:40–52 10.1038/s41582-018-0101-0 PMC8796686 30451971

[7] El-Hattab AW, Adesina AM, Jones J, Scaglia F (2015) MELAS syndrome: clinical manifestations, pathogenesis, and treatment options. Mol Genet Metab 116:4–12 10.1016/j.ymgme.2015.06.004 26095523

[8] Engelen M, Abbink TEM, Salomons GS, van der Knaap MS (2021) Leukoencephalopathy with Brain Stem and Spinal Cord Involvement and Lactate Elevation. 2010 May 25 [updated 2021 Feb 18]. In: Adam MP, Bick S, Mirzaa GM, Pagon RA, Wallace SE, Amemiya A (eds.) GeneReviews® [Internet]. University of Washington, Seattle; 1993–2026 20506600

